# How Immunization Information Systems Inform Age-Based HPV Vaccination Recommendations in the United States: A Mixed-Methods Study

**DOI:** 10.3390/vaccines13070716

**Published:** 2025-06-30

**Authors:** Nadja A. Vielot, Isabelle K. Bucklin, Kristy Westfall, Deanna Kepka, Gregory Zimet, Sherri Zorn

**Affiliations:** 1 Department of Family Medicine, University of North Carolina at Chapel Hill, Chapel Hill, NC 27599, USA; 2 School of Medicine, University of North Carolina at Chapel Hill, Chapel Hill, NC 27599, USA; isabelle_keim@med.unc.edu; 3 Association of Immunization Managers, Rockville, MD 20850, USA; kwestfall@immunizationmanagers.org; 4 Huntsman Cancer Institute and College of Nursing, University of Utah, Salt Lake City, UT 84112, USA; deanna.kepka@hci.utah.edu; 5 Department of Pediatrics, Indiana University School of Medicine, Indianapolis, IN 46202, USA; gzimet@iu.edu; 6 Washington State HPV Free Task Force, Tumwater, WA 98501, USA

**Keywords:** human papillomavirus, immunization information system, vaccination, adolescents

## Abstract

**Background**: Immunization information systems (IISs) in the United States forecast vaccine due dates, which can inform when providers recommend vaccines to patients. IIS forecasting for HPV vaccination at 9 years, the minimum age of licensure, and when vaccination is likely most effective is not documented or well-understood. **Methods**: We documented characteristics of HPV vaccination forecasts in jurisdictional IISs through Internet searches and requests to immunization program managers. Next, we conducted focus groups with stakeholders from seven jurisdictions to elucidate their processes for determining and implementing HPV vaccination forecasts. **Results**: Forecast data were available from 49 out of 64 CDC-funded jurisdictions, of which 14 (29%) recommended HPV vaccination at age 9 and 35 (71%) recommended HPV vaccination starting at ages 11 through to 15. Jurisdictions that recommended HPV vaccination at age 9 cited the positions of the American Cancer Society and American Academy of Pediatrics and reported little or no provider opposition to this recommendation. Jurisdictions reported variable flexibility in programming their forecasts. Those that changed their HPV vaccination forecast from 11 to 9 years did so easily while some experienced limitations. Other jurisdictions adhered strictly to the CDC’s routine recommendation at age 11–12 years and would only update the forecast in tandem with updated CDC guidance. The impact of IISs and electronic health record interoperability on how providers view and utilize IIS forecasting is unclear. **Conclusions**: Jurisdictions can share best practices for forecasting at 9 and future studies can evaluate the effects of forecasting age on the vaccination rates, providing evidence for nationwide vaccination recommendations.

## 1. Introduction

Immunization information systems (IISs) are electronic databases that track immunizations administered by providers within a defined jurisdiction. Sixty-four jurisdictions in the United States (50 states, the District of Columbia, 6 cities, 8 territories) receive U.S. Centers for Disease Control and Prevention (CDC) funding to implement an IIS according to the Public Health Service Act 317b [1,2]. Jurisdictional IISs are generally managed by the immunization branches of their departments of health, and guided by local policies for vaccination reporting, data sharing, and implementing CDC recommendations for vaccination. IISs are used by jurisdictional health department staff to monitor the vaccination rates; by clinicians to check the patients’ vaccination status; and by community members to provide vaccination records as per the school or work requirements. There are various IIS platforms available to jurisdictions with unique functionality, causing variability in IIS programming [1,3,4,5]. The three most used IIS platforms include STChealth (Phoenix, AZ, USA), Envision (Greenwood Village, CO, USA), and the Wisconsin Immunization Registry (WIR, Madison, WI, USA), all of which are used across multiple jurisdictions. Some jurisdictions employ “homegrown” systems that were designed and built locally to that jurisdiction’s specifications and in accordance with its vaccination policies and priorities [6].

IISs employ clinical decision support for immunization, also known as vaccination forecasting, which translates the clinical language of CDC recommendations for vaccination into technical logic [7]. IISs can determine the due dates for immunizations based on age, allowing providers to order and administer vaccines, schedule future vaccinations, and identify under-vaccinated patients who require follow-up. IISs are also capable of real-time, bidirectional data exchange with electronic health record (EHR) systems, providing the option to conveniently access IIS forecasting data through the EHR [8,9,10,11]. These IIS features maximize efficiency in clinic encounters and assist providers in ensuring that their patients are up to date on the recommended vaccinations.

The CDC’s Advisory Committee on Immunization Practice (ACIP) routinely recommends two doses of human papillomavirus (HPV) vaccine to 11- and 12-year-olds, with an option to start at age 9 [12,13]. However, while the U.S. Department of Health and Human Services Healthy People 2030 goal is for 80% of adolescents aged 13–15 years to receive all recommended vaccine doses [14], the national up-to-date HPV vaccination coverage is only 57% as of 2023 [15]. A provider’s recommendation is a strong predictor of initiating and completing the HPV vaccination series [16,17,18], and recent evidence suggests that recommending HPV vaccination at age 9, the earliest opportunity, instead of age 11, can lead to higher rates of up-to-date vaccination by age 13 [19,20,21]. Starting the conversation earlier provides more opportunities to vaccinate, including in the event of access issues or disruption of care, allows more time for providers to educate caregivers about HPV vaccination, and might reduce the pressure to have conversations around sexual activity [22]. Consequently, the American Cancer Society (ACS) and the American Academy of Pediatrics (AAP) recommend routine HPV vaccination starting at age 9 [23,24].

Disparate age-based forecasts for HPV vaccination in jurisdictional IISs could influence how HPV vaccination recommendations are made by providers and lead to geographic disparities in HPV vaccination rates, but these data had not previously been compiled. We conducted this mixed-methods study to (1) document the age at which jurisdictional IISs forecast HPV vaccination, and (2) describe how jurisdictional immunization programs determine and implement HPV vaccination forecasts. The findings can guide jurisdictions on the capabilities of IISs for forecasting HPV vaccination, and unique insights from IIS stakeholders can inform clinical practice and national recommendations.

## 2. Materials and Methods

First, we documented the age ranges of HPV vaccination forecasts by searching the health department webpages of the 64 CDC-funded jurisdictions for informational materials that showed examples of the IIS forecast interface with a test patient. We contacted immunization program managers to request HPV vaccination forecast data that were not available online. Data were collected until June 2025.

Next, we conducted focus group discussions with IIS stakeholders from jurisdictions purposively identified by the study team and the Association of Immunization Managers as representing various regions, IIS platforms, and HPV vaccination forecast characteristics. We sent email invitations to immunization program leadership groups asking them to nominate participants who were knowledgeable about IIS implementation including health department staff (e.g., immunization branch managers, epidemiologists, public health nurses) and external stakeholders (e.g., providers, community education, and outreach workers). Given that each jurisdiction has a different organization of the immunization branch and IIS staffing, we allowed for a variety of focus group compositions with respect to participant roles in IIS decision-making and execution. Jurisdictions that agreed to participate largely had existing collaborative relationships with the investigators. To prevent deductive disclosure of the participants, we do not report here the participants’ jurisdictions or personal characteristics.

### 2.1. Measures

We reviewed the published literature and government agency reports to determine what was already known about jurisdictional IIS development and management and identified underrepresented themes that we wished to capture in our study [1,4,5,25]. We developed a discussion guide to probe about technical aspects of the IIS including the system and vendor used; perceived benefits and limitations of IIS platforms; decision-making for and customizability of IIS functions; use of IIS for vaccination forecasting; and IIS use in clinical settings for recommending vaccines. The study principal investigator and a note-taker conducted and recorded the focus groups virtually using Zoom teleconferencing software version 6.0 (Zoom Communications, San Jose, CA, USA). We securely transferred the video files to a professional transcriptionist who prepared transcripts for thematic analysis.

### 2.2. Analysis

We developed an initial codebook based on prompts from the guide, and two reviewers (N.A.V. and I.K.B.) independently coded the same transcript to assess the coding consistency using Dedoose Version 9.0.17 (SocioCultural Research Consultants, LLC; Los Angeles, CA, USA). The initial interrater reliability according to Regier’s criteria was very good (kappa = 0.63) and increased (kappa = 0.78) on review of the first transcript and resolution of discrepancies, after which I.K.B. coded the remaining transcripts. We refined and expanded the codebook as new themes emerged from the data and conducted thematic analysis by summarizing the most identified themes and presenting illustrative quotations.

This study was not considered as human subjects research (i.e., not “a systematic investigation designed to develop or contribute to generalizable knowledge” as per the U.S. Department of Health and Human Services Policy for Protection of Human Subjects, 45 CFR Part 46) and was exempted from review by the Institutional Review Board of the University of North Carolina at Chapel Hill (IRB #: 23-1576). In lieu of collecting informed consent from the participants, we provided them with a description of the research question, data collection procedures, and intended use of the data prior to recording the focus group discussions.

## 3. Results

### 3.1. Survey of HPV Vaccination Forecasts

**Figure 1:**
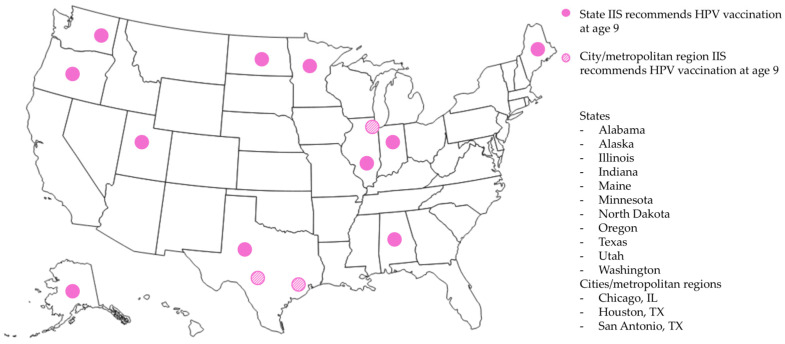
U.S. jurisdictions recommending HPV vaccination at age 9 in IISs as of June 2025.

Between September 2023 and June 2025, we obtained data on HPV vaccination forecasts from 45 states and 4 territories (Table 1). Six jurisdictions (12%) offered one age recommendation for HPV vaccination, whereas forty-three (88%) offered a range of eligible ages (Table 1). Age ranges were based largely on the CDC recommendations for routine (9–12 years), catch-up (13–26 years), and shared decision-making (27–45 years) vaccination. Forty-two jurisdictions specified a minimum age for HPV vaccination, of which forty specified nine years as the minimum. Two jurisdictions specified 12 and 15 years as the minimum ages in contrast to the national recommendations. The overdue age, when offered (*n* = 34), was typically age 13 (*n* = 30), in accordance with the CDC’s recommendation for up-to-date routine adolescent vaccination. All jurisdictions provided a “recommended” age or date for HPV vaccination, which was 11 years in 33 jurisdictions (67%). As of June 2025, 14 jurisdictions (29%), including Alabama; Alaska; Chicago, IL; Houston, TX, Illinois; Indiana; Maine; Minnesota; North Dakota; Oregon; San Antonio, TX; Texas; Utah; and Washington, indicated 9 years as the recommended and the minimum age (Figure 1).

### 3.2. Focus Groups on IIS Implementation

#### 3.2.1. Overview of Participating Jurisdictions

In March 2024, we held focus groups with stakeholders from seven jurisdictions, with three to seven participants per group (33 participants total). Most participants were IIS managers, public health nurses, immunization branch leaders, immunization coordinators, and public health professionals (Table 2). While the composition of each focus group differed, all seven focus groups included a full-time or acting IIS manager and four included an immunization branch leader. Jurisdictions used the STChealth (*n* = 3), Envision (*n* = 1), WIR (*n* = 2), and homegrown (*n* = 1) platforms. The main themes of discussion included the customizability of IIS; decision-making around IIS functions; historical changes made to HPV vaccination forecasts; and IIS interoperability with EHR.

#### 3.2.2. Customizability of IISs

The customizability of IIS functions, including vaccination forecasts, depended largely on the platform. Jurisdictions using STChealth reported an ease of customization at the jurisdictional level. Conversely, Envision makes IIS enhancements across its user consortium, thus limiting the jurisdictional ability to customize its IIS.

*“So they’ve really gone into a model of they need to evaluate this as a consortium as a whole and determine whether or not it’s a change that is going to benefit the whole consortium. So sometimes we don’t even have a say in that decision-making.”*

Jurisdictions using WIR have access to code that they can modify according to their needs.

*“Wisconsin owns the base code and the license overall, but … we can make the changes that we need that make sense for our jurisdiction, but we need to be willing to share those with others.”*

One jurisdiction used a homegrown system with a vendor to oversee the forecasting feature. Updates to forecasts were made in collaboration with STChealth, whereas others were made internally.

*“We can make changes more quickly instead of a vendor. And the look and feel, and everything, is totally customizable.”*

#### 3.2.3. Decision-Making Around IIS Functions

In most jurisdictions, immunization policy decisions were made by the health department or immunization branch leadership, whereas decisions about IIS implementation and management were made by the immunization branch and IIS staff. Some jurisdictions reported obtaining IIS policy approval through a vaccine advisory committee. Four jurisdictions reported receiving input from immunization partners for promoting vaccination.

*“We do have close coordination with our Chapter of the AAP, with our Medicaid agencies, [the jurisdiction immunization coalition]. We have all of our Vaccines for Children (VFC) providers that have certain requirements in order to be VFC providers. We work closely with pharmacies as well. There are lots of different entities that we hear feedback from and engage with that help to inform policies and things.”*

All jurisdictions cited CDC recommendations as the primary sources guiding vaccination forecasting. Jurisdictions that forecasted routine vaccination at age 9 additionally cited the AAP and ACS guidance and suggestions from providers.

*“There was lots of different advocacy groups coming out pushing 9. …Multiple people had come to us saying, hey, can this change? Providers were asking for it, which was pretty great.”*

The other jurisdictions agreed that they would update the HPV vaccination forecast only if the CDC updated its routine recommendation to age 9, despite personal or jurisdictional preferences.

*“I like standards, I like for the forecaster and for the system to behave according to ACIP recommendations pretty strictly. I personally wish the routine recommendation was at 9, that would clear up a lot of confusion.”*

#### 3.2.4. Changes to HPV Vaccination Forecast

In recent years, three jurisdictions have updated their forecast to indicate 9 years as the recommended age for HPV vaccination, and one added the option to vaccinate at age 9. In one jurisdiction, concerns about low HPV vaccination rates drove this change:

*“…we still saw significant gaps between HPV initiation and the Tdap and meningococcal vaccinations and also significant gaps between HPV completion and HPV initiation… That started broader conversations for us about what we can do to increase both initiation and completion rates. And I think that strategically we were also seeing it as starting earlier at age 9 gives providers more time to achieve completion by age 13.”*

In another jurisdiction, the forecast update responded to updated clinical recommendations:

*“We changed all our media and followed what the ACS was doing and changed our recommendation to 9. And we felt like, because we were recommending at 9, we should probably make that change [to the IIS]. And we actually did take it to the vaccine advisory board, and we had to get approval from them to do it.”*

In a third jurisdiction, advocacy from HPV vaccination stakeholders encouraged providers to recommend vaccination at age 9 and facilitated the associated forecast update.

*“So we really utilized our HPV Free Task Force to start to spread that message that people were allowed to start at 9. And because the actual recommendation from [the Department of Health] had not actually made that formal change in IIS, we really just shared this message as, this is an allowable thing.”*

*“But we said what if it [IIS] actually said that they’re due at 9 … would that make a difference? And we made sure that we had widespread support for it in our immunization community, and then it got brought to the vaccine advisory committee: let’s discuss this. Is there enough support behind this?”*

These three jurisdictions took care not to appear in opposition to the CDC recommendations and educated providers on the implications of the forecast update.

*“…How would we do the wording so that we don’t lose CDC funding, break some kind of contract? It’s just like, we don’t want to make an enemy, so how do we do this in concert with them, so that we have their approval? …So we were able to basically say we’re swimming with the CDC, we’re not going against them, we’re all going in the same direction.”*

*“…There can be some provider anxiety because it feels very different to them, but I think that our provider communication plan really acknowledges that difference in how to explain this to providers in a way that doesn’t feel like this is a massive switch but an expansion upon the work that they are already implementing.”*

The response from providers to the age 9 forecast update was largely favorable in all three jurisdictions:

*“…the culture has definitely shifted. I was in a meeting yesterday where everybody was talking about, ‘Oh, it’s a lot easier, and, you know, I didn’t think it would be.’”*

*“We have actually partnered with a provider who has been a long-time champion of vaccinating starting at age 9, well before any of this conversation, as it was up to a clinician’s discretion to be able to vaccinate at age 9. And so he has done a statewide webinar that was pretty well-received and is helping us think about how we frame out that clinician educational piece of implementation at a clinic level of the strategy of 9.”*

In one other jurisdiction, the update to indicate age 9 as “optional” in 2022 was a response to requests from providers and local health departments.

*“When we were made aware that we could have it as optional starting at age 9, because it used to say, “Not yet due until age 11,” … we talked about it, and we said, ‘Yeah, we definitely want to do that’….”*

Jurisdictions that made changes to their HPV forecast reported that the technical aspects were straightforward to make on their own.

*“Every state [that uses STC Health] has the option to override the CDC requirements or ACIP requirements….”*

*“…changing the earliest recommendation age for a vaccine schedule like that is super simple. I went in and moved it from 11 down to 9.”*

While updating the forecast did not require many resources, one jurisdiction reported other costs associated with updating the forecast.

*“And of course, then there was the cost of publicizing it, making a training video, and making the dashboard. There are those downstream costs to promoting it at age 9, for sure.”*

The Envision state reported that changes to the forecast are “locked down” and would have to be made by Envision throughout the user consortium. Furthermore, within the state, there was inconsistent support for recommending HPV vaccination at age 9 and limited viability of the idea at the time of the discussion.

*“We’d have to go through a pretty involved partner engagement process to look at the pros and cons of setting something in place like that here.”*

#### 3.2.5. Interoperability with EHR

All IISs performed bidirectional information exchange with EHR, allowing providers to see IIS forecast information in the EHR interface. Two jurisdictions reported that most IIS queries were made through EHR; one reported that less than 20% of providers accessed the IIS directly; another reported that half of the VFC providers made IIS queries through EHR and half directly accessed the IIS; and another reported that most users interfaced directly with the IIS.

*“…we print the forecast [from the IIS] and put it on the clipboard when we go in the room, and we look at the actual IIS forecast more than we’re actually looking at our EHR. And there’s historical reasons for that, because the EHR, when it was getting built with the recommendations in the EHR sometimes weren’t accurate, and so it didn’t build a ton of trust for pediatricians in navigating that system initially.”*

An unanswered question was how IIS information were interpreted and displayed in EHR, and which system was preferred by providers for assessing vaccination status.

*“So I talk to a lot of providers that look in both systems all of the time, maybe they don’t know that they have the functionality to view it in their EHR, maybe they don’t like the display, that [the IIS] is easier for them… So I would say there’s a lot of variation among workflows, too.”*

## 4. Discussion

IIS implementation varies across jurisdictions, with differences in the choice of platform and vendor, decision-making structures, the degree of IIS customizability within jurisdictions, and the forecast settings for HPV vaccination. While most IISs indicate 11 years as the recommended age with 9 years as the minimum age, 14 jurisdictions indicate 9 years as the recommended age. It remains to be seen how many jurisdictions will implement this strategy in the coming years, whether it will lead to increases in up-to-date vaccination, and whether the CDC will modify its routine recommendation accordingly.

Most jurisdictions implemented one of three IIS platforms, providing consistency in functionality within user groups. Users of two IISs reported having support from a consortium of users from other jurisdictions, allowing them to jointly troubleshoot technical issues and make technical requests on behalf of all users. Depending on the platform, the jurisdictions reported a variable level of flexibility in how they interpreted and implemented the vaccination forecasts. Many jurisdictions that we interviewed, though theoretically in favor of recommending HPV vaccination at age 9, wished to avoid perceived conflict with the CDC’s routine recommendation and programmed their forecasts accordingly. The wording of the CDC recommendation, which does not recommend routine HPV vaccination at age 9, may present a barrier for jurisdictions that wish to support vaccination at age 9. Currently, the ACIP is reviewing evidence in support of vaccination at age 9, but there is insufficient evidence to suggest that this change would be acceptable to providers and caregivers, and there are concerns that the change would undermine the adolescent vaccination platform in which HPV is co-administered with Tdap and meningococcal vaccines [26]. More data are needed on the relative benefits versus harms to support a change to the wording of the CDC recommendation, without which jurisdictional policies and practices are unlikely to implement the age 9 recommendation on a large scale.

Jurisdictions that indicate age 9 as the recommended age for HPV vaccination reported that the change was highly requested by providers, technically simple, and well-received by IIS users. There was very little reported criticism from providers, and this change is likely to be acceptable in other jurisdictions. Moreover, evidence from Washington state has shown vaccination initiation rates in 9- and 10-year-olds doubled within a year of changing the forecast age to 9 years [27]. Future studies will assess the impact of age 9 forecasts on HPV vaccination rates nationwide considering the mounting evidence that recommending HPV vaccination at age 9 leads to higher uptake and on-time completion. Furthermore, several studies have shown that providers perceive a greater parental acceptance of HPV vaccination at age 9 or 10, as sexual activity is not yet a topic of concern at younger ages [22,28,29]. As jurisdictions increasingly disseminate the age 9 recommendation for HPV vaccination, best practices can be shared across jurisdictions in a grassroots effort, and new opportunities to evaluate the impacts of this practice will contribute to evidence potentially in support of nationwide policy change.

More studies are needed to understand the relative utility of IIS forecasting compared with best practice guidance provided in the EHR, which can also forecast vaccination due dates and can be customized within and between healthcare systems [8,30,31]. We found that jurisdictions were highly varied in the degree to which users accessed the IIS directly versus querying the IIS through the EHR. Forecast data might be translated and displayed differently across EHR systems, potentially presenting forecast data in different and unintended ways. However, the focus group participants were largely not clinicians and could not comment on the differences between the IIS and EHR displays of the forecast data. Additionally, the wide variety of EHR systems in use nationwide makes it difficult to definitively or succinctly describe any discrepancies between the IIS and EHR forecasts. There is a need for future research in this area to identify the optimal use of health information technology for vaccination recommendations and potentially streamline conflicting recommendations between IIS and EHR. Considering the current wording of the CDC recommendation, it is likely that discrepancies in the preferred recommendation age will continue to exist.

Limitations of this study include the lack of comprehensive jurisdictional data on HPV vaccination forecasts and a limited number of jurisdictions providing qualitative data. We largely conducted focus group with jurisdictions with large populations, whereas smaller jurisdictions with less funding for IIS might have had different insights into priority settings for HPV vaccination forecasting. However, ours is the first assessment of jurisdictional IIS implementation that describes the diversity of HPV vaccination forecasts. This data can be useful to other jurisdictions interested in updating their forecasts and could be considered in discussions about future updates to the national HPV vaccination recommendations.

It remains to be seen whether forecasting HPV vaccination at age 9 influences how providers recommend HPV vaccination and the subsequent vaccination rates. Table 3 shows the CDC’s most recent estimates of up-to-date HPV vaccination coverage by IIS forecast age, but there is no clear trend to suggest that jurisdictions that forecast HPV vaccination at age 9 achieve a higher coverage than others. Longitudinal studies, including natural experiments that measure changes in vaccination rates before and after the implementation of age 9 forecasting, can indicate whether there is an association between the forecast and vaccination rates. However, multiple factors besides prompts from the IIS or EHR can influence vaccine recommendations and subsequent uptake including provider specialty and cost [29,32]. For example, insured children are more likely to receive HPV vaccination than uninsured children, and while uninsured or underinsured children in all jurisdictions are eligible to receive free vaccines through the VFC program, administrative costs still apply and could be prohibitive for low-income children. Future studies will have to assess the impact of IIS recommendations in the context of various other access and quality improvement initiatives to provide evidence for best clinical practice and for updated national recommendations.

Despite technological advancements in IIS functionality, a major limitation of jurisdictional IIS is the lack of an integrated national IIS or interoperability among existing IISs, which would reduce disparities in the implementation and utility of vaccination forecasts. An added benefit would be the ability to track individual vaccination records across jurisdictions for more accurate reporting of vaccination status and associated vaccination forecasting. A recent review of 264 global IISs showed that IISs in other countries are equally variable in function and often have similar limitations to IISs in the U.S. including the lack of a comprehensive national registry [33]. The U.S. has an opportunity to learn from and adapt best practices from other countries to streamline and strengthen its IISs, allowing for consistency in vaccination recommendations nationwide and potentially reducing the geographic disparities in vaccination recommendations and uptake.

## 5. Conclusions

Wide variations in IIS implementation across U.S. jurisdictions can cause inconsistencies in the dissemination of the CDC vaccine recommendations. The absence of a decisive age-based recommendation for HPV vaccination can potentially hinder timely vaccination efforts and exacerbate geographic disparities in uptake. Our findings suggest that streamlining the recommendation for HPV vaccination is largely feasible in existing IISs, but the current language of the CDC recommendation may discourage making recommendations at the earliest possible opportunity. By tracking the characteristics of the HPV vaccination recommendations across jurisdictions over time, we can provide evidence of increasing interest in age 9 HPV vaccination and its impact on vaccination rates, thereby contributing to nationwide policy decisions that provide the maximum support for this life-saving intervention.

## Figures and Tables

**Table 1:** Description of HPV vaccine forecast characteristics by jurisdiction (*N* = 49).

Characteristics	*n* (%)
Offers one age or date option for HPV vaccination	6 (12)
Offers a range of age or date options for HPV vaccination	43 (88)
Recommended age is 9 years	14 (29)
Recommended age is 11 years	33 (67)
Recommended age is 12 years	1 (2)
Recommended age is 15 years	1 (2)
Offers a minimum age or date (*n* = 42)	
Minimum age is 9 years	40 (95)
Minimum age is 12 years	1 (2)
Minimum age is 15 years	1 (2)
Offers an overdue age or date (*n* = 34)	
Overdue age is 12 years	1 (3)
Overdue age is 13 years	30 (88)
Overdue age is 14 years	1 (3)
Overdue age is 15 years	2 (6)

**Table 2:** Self-reported job titles of the focus group participants (*n* = 33).

Job Title	n *
IIS manager	6
Public health nurse consultant	4
Immunization branch director/deputy director/supervisor	5
Immunization coordinator	4
Public health educator/manager/director	2
CDC field designee/public health advisor	2
Epidemiologist	2
Data quality/quality improvement coordinator	2
Business analyst	2
Clinical application/services coordinator *	2
Pediatrician *	2
Cancer control program coordinator	1
Vaccine systems and support manager	1
Health promotion specialist	1

* Some participants reported multiple job descriptions.

**Table 3:** Jurisdictional up-to-date HPV vaccination coverage ^1^ by IIS forecast age ^2^.

Age 9 Forecast		Age ≥ 11 Forecast	
North Dakota	78.5 (69.3 to 85.6)	Rhode Island	80.4 (68.8 to 88.3)
Illinois—City of Chicago	75.6 (65.0 to 83.8)	Massachusetts	80.2 (72.8 to 86.0)
Texas—City of Houston	68.1 (57.8 to 76.8)	Hawaii	68.1 (59.7 to 75.5)
Oregon	65.3 (56.3 to 73.4)	South Dakota	65.6 (55.8 to 74.2)
Minnesota	65.0 (56.4 to 72.6)	Colorado	65.2 (57.0 to 72.6)
Illinois	63.0 (56.8 to 68.9)	Michigan	64.9 (54.3 to 74.2)
Alabama	59.1 (51.1 to 66.7)	New Mexico	64.3 (55.0 to 72.6)
Maine	58.8 (50.7 to 66.5)	Wisconsin	63.6 (55.6 to 70.9)
Washington	58.6 (48.4 to 68.1)	Maryland	63.6 (55.0 to 71.4)
Utah	58.4 (47.4 to 68.6)	Iowa	63.2 (53.2 to 72.2)
Indiana	57.1 (47.5 to 66.3)	Connecticut	63.1 (49.7 to 74.8)
Texas	54.3 (45.7 to 62.6)	New York State	61.7 (55.2 to 67.8)
Texas—Bexar County	52.6 (43.5 to 61.5)	Vermont	61.5 (50.3 to 71.7)
Alaska	47.5 (37.7 to 57.6)	New Hampshire	61.3 (51.9 to 70.0)
		North Carolina	61.2 (51.3 to 70.2)
		Ohio	60.6 (50.7 to 69.7)
		Nebraska	58.4 (48.8 to 67.5)
		Florida	58.4 (48.1 to 68.1)
		Louisiana	58.1 (48.5 to 67.1)
		Arizona	57.6 (48.2 to 66.5)
		South Carolina	56.3 (47.4 to 64.9)
		California	56.1 (45.7 to 66.0)
		Virginia	56 (44.5 to 66.9)
		Tennessee	54.2 (42.9 to 65.1)
		Missouri	50.4 (39.4 to 61.4)
		Idaho	50.0 (40.6 to 59.4)
		West Virginia	48.2 (40.2 to 56.3)
		Wyoming	47.9 (38.9 to 56.9)
		U.S. Virgin Islands	45.9 (28.5 to 64.4)
		Kentucky	44.5 (36.0 to 53.4)
		Nevada	44.1 (35.2 to 53.4)
		New Jersey	42.7 (34.9 to 50.9)
		Georgia	40.1 (29.3 to 52.0)
		Oklahoma	36.1 (27.6 to 45.6)

^1^ Up-to-date (received ≥2 HPV vaccines doses by age 13) HPV vaccination coverage among adolescents aged 13–15, 2023 NIS Teen Survey (https://www.cdc.gov/teenvaxview/interactive/index.html, accessed on 19 June 2025). ^2^ As of June 2025.

## Data Availability

Quantitative data were collected from publicly available Internet sources. Qualitative data were anonymized to protect the identity of the focus group participants and are available only by request and with approval from the participants.

## Abbreviations

The following abbreviations are used in this manuscript:

AAP	American Academy of Pediatrics
ACIP	Advisory Committee on Immunization Practices
ACS	American Cancer Society
CDC	U.S. Centers for Disease Control and Prevention
EHR	Electronic health records
HPV	Human papillomavirus
IIS	Immunization information system
Tdap	Tetanus-diphtheria-acellular pertussis vaccine
